# Correlation between quadriceps activation and proprioception in patients with knee osteoarthritis

**DOI:** 10.3389/fbioe.2025.1735163

**Published:** 2026-01-30

**Authors:** Meiping Yang, Zhilin Ge, Zibo Gao, Xiaolong Zeng, Yongjin Li, Dingkun Lin, Yu Hou

**Affiliations:** 1 The Second Clinical Medical College, Guangzhou University of Chinese Medicine, Guangzhou, China; 2 Department of Orthopedics, The Second Affiliated Hospital of Guangzhou University of Chinese Medicine, Guangzhou, China; 3 Department of Orthopedics, Guangdong Provincial Hospital of Traditional Chinese Medicine, Guangzhou, China; 4 Guangzhou Key Laboratory of Cervical Mechanobiology, Guangzhou, China; 5 Chinese Medicine Guangdong Laboratory, Zhuhai, China

**Keywords:** knee, osteoarthritis, proprioception, quadriceps, surface myoelectricity

## Abstract

**Objective:**

To investigate the correlation between the activation status of quadriceps muscle and the proprioceptive accuracy in patients with knee osteoarthritis (KOA).

**Methods:**

We included 43 outpatients diagnosed with KOA and 45 health subjects from October 2024 to May 2025. The correlation between the absolute error in knee Joint Position Reproduction (JPR) and the root mean square (RMS) and median frequency (MDF) of quadriceps surface electromyography during walking and stair climbing was analyzed.

**Results:**

The JPR of 45° in KOA patient is significantly higher than health subjects. There was no significant difference in 30° and 60° JRP between the two groups. While walking, there is no significant difference of the sEMG data in two group. While going up stair, the RMS of LVM and RVM in KOA group had increased activation compared with the health subjects. In KOA group, while going up stair, the JPR absolute error angle of 30° was negatively correlated with the MDF of RVL, the JPR absolute error angle of 45° was positively correlated with the RMS of RVM, and the JPR absolute error angle of 60° was positively correlated with RMS of LVM and RVM. There are no significantly correlation in JPR absolute error angle and the surface electromyography of the quadriceps muscle while walking and in health control group. Partial correlation analysis was performed on the aforementioned correlated indicators with gender included as a control variable, and the results indicated that in women group, the JPR absolute error angle of 60° was positively correlated with RMS of LVM and RVM. In KOA group, the RMS of LVL, RVL and RVM in walking was positively correlated with the RMS of LVL, RVL and RVM in going up and down stair, and the MDF of LVL, RVL and RVM in walking was positively correlated with the MDF of LVL, RVL and RVM in going up and down stair. In health control group, the MDF of LVL, RVL and RVM in walking was positively correlated with the MDF of LVL, RVL and RVM in going up and down stair.

**Conclusion:**

This study indicated that during stair climb, KOA patients display a unique pattern of elevated RMS of the VM. Notable relationships were identified between the absolute error angles of JPR and sEMG parameters of VM. The pronounced positive correlations in muscle activation patterns between walking and stair upward and downward activities indicate a fixed, task-invariant neuromuscular approach in KOA patients, possibly resulting from compromised sensorimotor integration due to proprioceptive impairments. These findings offer new insights into the pathogenesis of KOA from a neuromuscular-sensory viewpoint, emphasizing the need for future rehabilitation therapies to concurrently target proprioceptive accuracy and the enhancement of muscle activation patterns.

## Introduction

Knee osteoarthritis (KOA) is a progressive disorder marked by a disparity between the degradation and regeneration of joint components (Hunter and Bierma-Zeinstra, 2019). The decline in motor function of the knee joint is correlated not only with localized tissue injury and discomfort but also with reduced muscle strength surrounding the knee, changes in muscle activation patterns, compromised proprioceptive accuracy, and weakened neuromuscular control. Impaired joint proprioceptive accuracy has been suggested as a possible local factor influencing the onset and advancement of KOA. Moreover, in individuals with KOA, there is an imbalance in muscular strength, proprioception, biomechanics, and postural stability of the lower extremities, with proprioception and muscle strength recognized as strongly linked variables (Salamanna et al., 2023; Khokhlova et al., 2024; Zeng et al., 2022; Yang et al., 2024).

Proprioception and muscular activation patterns are intricately associated with neuromodulation. The proprioceptive feedback loop, characterized by the awareness of the limb’s absolute position and its positional alterations in space via muscle spindles, tendon organs, and other sensory receptors, furnishes the brain with crucial information for the continuous biological coordination and regulation of muscle activity, facilitating prompt movement adjustments. Thus, diminished proprioceptive precision can adversely affect the velocity, extent of muscle activation, and muscular strength. KOA lesions modify the activation sequence of lower limb muscles during different movements, leading to delayed activation of the quadriceps and alternate delays in the activation of the lateral and medial femoral muscles. The extent of muscle activation indicates the excitability of neuromuscular function (Taniguchi et al., 2024; Zhu et al., 2024).

Atrophy of the quadriceps, especially the vastus medialis (VM), presents as decreasing muscle strength, reduced muscle circumference, and decreased cross-sectional area of muscle fibers (Yamauchi et al., 2020; Cunha et al., 2019). Delayed quadriceps activation occurs during ambulation and stair ascent, accompanied by heightened synergistic muscular co-contraction, resulting in muscle exhaustion (Taniguchi et al., 2024; Chen et al., 2023; Rashid et al., 2022). The correlation between aberrant muscle activation and proprioceptive accuracy in knee osteoarthritis remains undefined. The muscle contractions in the elderly necessitate increased capacity and a higher frequency of nerve impulses (Sanders et al., 2019; Iijima et al., 2020). Insufficient muscle mobilization or movement angle at the knee joint leads to aberrant walking patterns in the elderly, drastically diminishing their balance and landing absorption capabilities, hence markedly increasing the risk of falls. Elucidating the relationship between quadriceps femoris muscle activation and proprioceptive dysfunction may provide a foundation for investigating the underlying mechanisms through which quadriceps femoris exercise exerts its effects in knee osteoarthritis. This study aims to concurrently assess the muscle activation status of the quadriceps and the proprioceptive accuracy of the knee joint in patients with KOA, while examining the link between muscle activation and proprioceptive accuracy. We hypothesized a favorable connection between quadriceps muscle activation and proprioceptive accuracy.

## Methodology

### Patient inclusion

Patients with KOA who attended the orthopedic outpatient clinic at the Guangdong Provincial Hospital of Traditional Chinese Medicine from October 2024 to May 2025 were included. Inclusion criteria of KOA patients are as follow: ① Diagnosis of KOA according to the revised criteria established by the American College of Rheumatology; ② Age between 55 and 75 years; ③ Kellgren-Lawrence (KL) classification of the affected knee ranging from Ⅲ to Ⅳ; ④ Unilateral KOA; ⑤ Voluntary participation in the study with a signed informed consent form. Exclusion criteria: ① Patients with knee trauma, prior surgery, tumors, tuberculosis, osteomyelitis, or similar conditions; ② Patients with rheumatoid arthritis, gouty arthritis, infectious arthritis, or pigmented villous nodular synovitis; ③ Patients with severe liver or renal insufficiency, serious cardiovascular disease, diabetes mellitus, Parkinson’s disease, Alzheimer’s disease, or psychiatric disorders; ④ Patients experiencing significant loss of exercise capacity due to factors other than KOA. The healthy control group comprises individuals without the condition, who are matched with the KOA group in terms of age, gender, and demographic background. This study was reviewed and approved by the Clinical Trial Ethics Committee of the Guangdong Hospital of Traditional Chinese Medicine (No. KY2021173).

### Surface electromyography (sEMG) data of the quadriceps muscle

The 8-channel distributed wireless electromyography acquisition system (Shenzhen Runyi Taiyi Technology Co., Ltd.) was utilized for data collection, with a sampling frequency of 2,000 Hz. Subjects were prepared by cleaning the skin of both lower limbs with 75% alcohol, and any hair in the areas of electrode placement was shaved. Surface electrodes were affixed to the following muscles in both lower limbs: the vastus lateralis (VL), located at the highest point of the muscle belly in the lower third of the line connecting the lateral edge of the patella and the anterior superior iliac spine; and the VM, placed at the midpoint of the line connecting the medial edge of the patella and the anterior superior iliac spine. The electrodes were positioned on the most prominent part of each muscle belly, aligned with the direction of the muscle fibers. Subjects were instructed to remain barefoot, and the testing procedure was thoroughly explained to them prior to the commencement of the test to ensure their understanding and comfort.

#### Quadriceps reference voluntary contraction (RVC) standardization

Participants were instructed to perform bodyweight squats to their maximum comfortable depth under standardized conditions. A three-dimensional motion capture gait system (Opti_Knee, Innomotion Inc., Shanghai, China) was used to determined the longitudinal axis of the femur and the longitudinal axis of the tibia create a 90-degree angle, the body inclines forward, and the center of gravity is situated at the base of the thumb on the anterior foot. The subjects maintained the squat position for 3 s, then stood up straight (Korak et al., 2020; Ball and Scurr, 2013). This process was repeated three times to determine the RVC sEMG. In this study, requesting KOA patients to undertake the Maximum Voluntary Contraction (MVC) test may induce pain, hindering their ability to apply true force. Furthermore, it poses significant hazards, including the potential for secondary injuries, and some participants may struggle to finish the assessment, leading to inaccurate results. In these instances, RVC provides a safer, more pertinent, and more viable alternative.

#### Treadmill walking

Participants stood still on the treadmill and adjusted the speed between 1 km/h and 3 km/h according to their habitual walking speeds. After 10 s of stable walking, the sEMG data of quadriceps and knee joint velocity data was collected for about 15 s (15 walking gait cycles) for both knee joints. A synchronizer was employed to simultaneously collect sEMG data alongside motion capture gait system data.

#### Walking up and down steps

Subjects stood naturally on the ground with their legs relaxed. Upon hearing the walking command, the subject began to ascend the steps, first lifting the right thigh, followed by the left thigh, and then the right thigh again, taking one step at a time for a total of three steps. After reaching the third step, the subject remained still for one second. Upon hearing the walking command again, the subject then descended the steps, first lowering the right thigh, followed by the left thigh, and finally standing on a flat surface. It is important to maintain an upright trunk throughout the process of ascending and descending the steps. Collect the data of going up and down the stairs three times. A synchronizer was also employed to simultaneously collect sEMG data alongside motion capture gait system data.

#### Signal processing

MATLAB analysis software was employed to process the original sEMG signals from the medial femoris and lateral femoris. The original electromyography signal is subjected to denoising via a 20–450 Hz bandpass Butterworth filter, followed by rectification and smoothing for preprocessing. The phase for walking we analyzed were Support phase of the gait cycle (0%–60%). This phase are significant because they correspond to critical phases of the gait cycle, each reflecting different biomechanical demands and kinematic behaviors. In the task of ascending and descending stairs, the limb that initiates the movement on the stairs is defined as the leading phase, while the limb that follows subsequently is referred to as the trailing phase. MATLAB analysis software was then employed to derive the amplitude values for all exercise cycles, determine the time range of each signal cycle based on the raw signals, and identify the Root Mean Square (RMS) and the Median Frequency (MDF) for each muscle within the Support phase of the gait cycle (0%–60%) and leading phase. Additionally, the RMS were normalized [RMS(walking or up stair)/RMS(RVC)] using data from the RVC test.

### Absolute error angle of knee joint position reproduction (JPR)

The YTK-E Continuous Passive Motion (CPM) system was utilized for the assessment. Prior to the evaluation, the patient donned loose clothing and exposed the affected knee, assuming a supine position to relax. The affected lower limb was positioned on the CPM device, which was adjusted to ensure that the center of flexion and extension of the CPM aligned with the lateral condyle of the femur. Once the patient was prepared, a blackout blindfold was applied to eliminate visual distractions, and the passive reproduction test for the affected side was conducted. The patient was informed about the test procedure, with knee flexion set at 0° as the starting position. The subject’s limb was then passively moved at a constant speed of 2°/s to the target angles of knee flexion (30°, 45°, and 60°) for a duration of 5 s before returning to the starting position. Subsequently, the knee was passively extended at the same speed of 2°/s, with the subject’s eyes closed, and the movement was halted once the limb reached the target angle. Each subject underwent a pretest to familiarize themselves with the procedure and minimize errors. The absolute value of the difference between the patient’s self-perceived positioning angle and the target angle was recorded, and the average was calculated after three trials. Half an hour after the initial measurement, a second researcher independently performed the same assessment on the subjects (Horváth et al., 2023). The mean of the two sets of measurements was computed, and the inter-rater correlation was evaluated to assess the reliability and consistency of the repeated measurements in the experiment. A smaller absolute difference indicated greater accuracy in the subject’s proprioceptive awareness of positional restoration. The angles of 30°, 45°, and 60° were selected as target positions for the JPR test, as they encompass the angular range required for essential daily activities, including walking, standing, stair climbing and descending, and sit-to-stand transitions. These angles also correspond to critical joint positions where significant changes in ligament tension occur and muscular coordination is actively engaged. As such, they effectively challenge the proprioceptive system and enable reliable differentiation between individuals with KOA and healthy controls.

### Statistical methods

Basic characteristics of the patients were described using means (standard deviation), n (%), or medians (interquartile ranges). The absolute error angle of the JPR for repeated test reliability by the same tester was expressed using the Pearson correlation coefficient. Correlation analysis was employed to determine the relationship between the absolute error angle of the JPR and the sEMG data of the medial and lateral femoral muscles across support phase of the gait cycle and leading phase of the ascending stairs. Partial correlation analysis was employed to examine the correlation while controlling for the potential confounding effect of gender. Additionally, gender-stratified analysis were conducted to assess the robustness and consistency of the findings across subgroups.

## Results

### Basic characteristics

In KOA patient group, 43 patients were included, 38 were female and 5 were male, with a mean age of 65.83 ± 7.58 years, and a body mass index (BMI) of 23.49 ± 3.21 kg/m^2^. Among the patients, there were 25 cases of left knee involvement and 18 cases of right knee involvement. The distribution of cases according to the Kellgren-Lawrence (K-L) grading system was as follows: 13 cases of grade Ⅲ, and 30 cases of grade Ⅳ, the mean VAS score was 2.32 ± 1.73, and the mean WOMAC score was 10.41 ± 8.90. In health subject group, 45 subjects were included, 40 were female and 4 were male, with a mean age of 64.87 ± 4.09 years, and a body mass index (BMI) of 23.37 ± 3.50 kg/m^2^.

### Absolute error angles in JPR

The distribution of the absolute error angle of JPR of the knee joints of two groups is detailed in Table 1. The Interclass Correlation Coefficient for the repeated tests at knee flexion angles of 30°, 45°, and 60° were 0.696, 0.794, and 0.772, respectively, demonstrating a significant correlation (P < 0.05). The JPR of 45° in KOA patient is significantly higher than health subjects (P < 0.05). There was no significant difference in 30° and 60° JRP between the two groups.

**TABLE 1:** Details of characteristic between KOA group and HC group.

Indicator	KOA group (n = 43) (x ± s)	HC group (n = 45) (x ± s)	p-value
Age (years)	65.83 ± 7.58	64.87 ± 4.09	0.45
Gender (F/M)	38/5	40/4	0.51
BMI (kg/m^2^)	23.49 ± 3.21	23.37 ± 3.50	0.86
Affected knee (right/left)	18/25	—	—
K- L grade (Ⅲ/Ⅳ grade)	13/30	—	—
VAS score	2.32 ± 1.73	—	—
WOMAC score	10.41 ± 8.90	—	—
30°JPR	5.24 ± 4.88	2.96 ± 2.23	0.10
45°JPR	9.53 ± 6.01	5.92 ± 4.07	0.02^*^
60°JPR	9.24 ± 6.06	6.30 ± 3.06	0.26
Walk_LVM_RMS(/RVC)	0.72 ± 0.74	0.52 ± 0.32	0.35
Walk_LVL_RMS(/RVC)	0.64 ± 0.27	0.61 ± 0.31	0.60
Walk_RVM_RMS(/RVC)	0.79 ± 0.37	0.74 ± 0.37	0.70
Walk_RVL_RMS(/RVC)	0.64 ± 0.26	0.69 ± 0.40	0.88
Stair_LVM_RMS(/RVC)	1.05 ± 0.32	0.79 ± 0.30	0.03^*^
Stair_LVL_RMS(/RVC)	0.84 ± 0.31	0.85 ± 0.25	1.00
Stair_RVM_RMS(/RVC)	1.01 ± 0.38	0.83 ± 0.33	0.04^*^
Stair_RVL_RMS(/RVC)	0.85 ± 0.28	0.79 ± 0.32	0.48
Walk_LVM_MDF	49.08 ± 19.69	48.92 ± 16.23	0.97
Walk_LVL_MDF	68.00 ± 24.58	66.15 ± 26.04	0.73
Walk_RVM_MDF	58.99 ± 22.32	54.72 ± 22.28	0.43
Walk_RVL_MDF	69.30 ± 21.52	64.41 ± 22.73	0.30
Stair_LVM_MDF	55.11 ± 13.97	55.36 ± 12.92	0.93
Stair_LVL_MDF	69.46 ± 22.61	64.47 ± 17.39	0.25
Stair_RVM_MDF	60.23 ± 22.07	59.88 ± 22.49	0.94
Stair_RVL_MDF	72.47 ± 19.71	69.54 ± 23.63	0.53

### sEMG of quadriceps in walking and walking up and down steps

The signal of LVM, LVL, RVM and RVL in walking and walking up and down steps were collected and processed. As shown in Table 1, while walking, there is no significant difference of the sEMG data in two group. While going up stair, the RMS of LVM and RVM in KOA group had increased activation compared with the health subjects.

### Analysis of the correlation between sEMG of quadriceps and JPR absolute error angle

**FIGURE 1:**
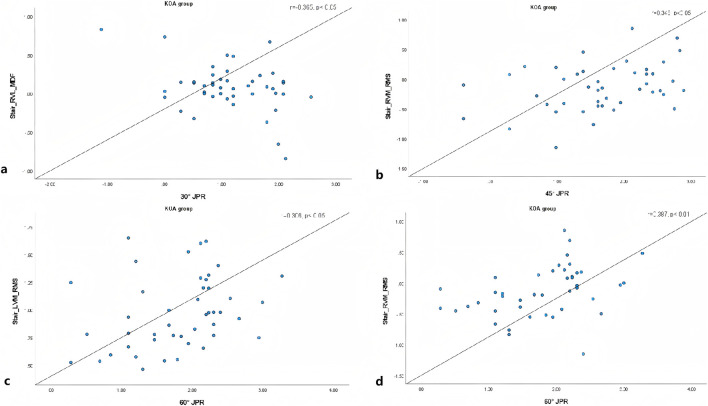
Correlation between quadriceps surface sEMG and JPR absolute error angle. Plot **(a)**: Stair_RVL_MDF vs.30°JPR (r = -0.365, p < 0.05). Plot **(b)**: Stair_RVM_RMS vs.45°JPR (r = 0.343, p < 0.05). Plot **(c)**: Stair_LVM_RMS vs.60°JPR (r = 0.368, p < 0.05). Plot **(d)**: Stair_RVM_RMS vs.60°JPR (r = 0.387, p < 0.01). Each plot includes a trend line.

The correlation between the surface electromyography of the quadriceps muscle and the angle of absolute error of the JPR in the KOA group is detailed in Figure 1. In going up and down stair, the JPR absolute error angle of 30° was negatively correlated with the MDF of RVL (r = −0.365, P < 0.05, 95% CI [−0.552, 0.002]), the JPR absolute error angle of 45° was positively correlated with the RMS of RVM (r = 0.348, P < 0.05, 95% CI [0.060, 0.582]), and the JPR absolute error angle of 60° was positively correlated with RMS of LVM (r = 0.362, P < 0.05, 95% CI [0.078, 0.593]) and RVM (r = 0.387, P < 0.05, 95% CI [0.105, 0.611]). There are no significantly correlation in JPR absolute error angle and the surface electromyography of the quadriceps muscle while walking and in health control group. Partial correlation analysis was performed on the aforementioned correlated indicators with gender included as a control variable, and the results indicated that in women group, the JPR absolute error angle of 60° was positively correlated with RMS of LVM (r = 0.301, P < 0.01, 95% CI [0.084, 0.491]) and RVM (r = 0.279, P < 0.05, 95% CI [0.060, 0.472]).

### Analysis of the correlation between quadriceps surface sEMG in walking and walking up and down steps

**FIGURE 2:**
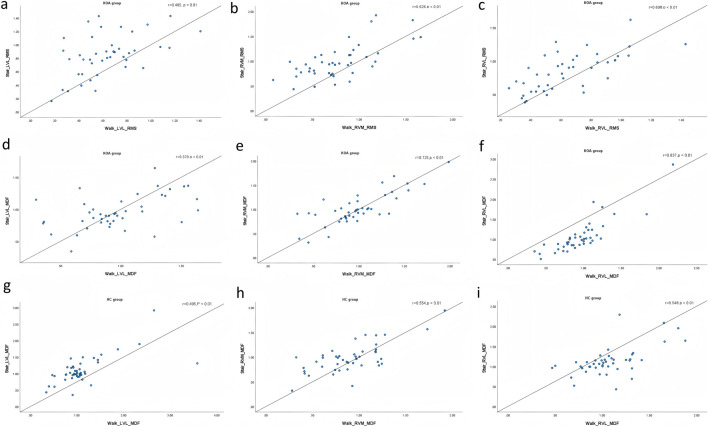
Correlation between quadriceps surface sEMG in walking and walking up and down steps. Each plot shows data points with a line arregression line and correlation coefficients. Panels are labeled **(a–i)**, depicting relationships between parameters like Walk_LVL, Walk_RVM, Walk_LVL, and Walk_RVL against Stair LVL, Stair RVM, and Stair RVL measurements. Correlation values and significance levels are displayed on each graph, indicating varying degrees of line arassociation.

As shown in Figure 2, in KOA group, the RMS of LVL, RVL and RVM in walking was positively correlated with the RMS of LVL, RVL and RVM in going up and down stair (r = 0.485, 0.698, and 0.626, respectively, P < 0.01), and the MDF of LVL, RVL and RVM in walking was positively correlated with the MDF of LVL, RVL and RVM in going up and down stair (r = 0.570, 0.837, and 0.725, respectively, P < 0.01). In health control group, the MDF of LVL, RVL and RVM in walking was positively correlated with the MDF of LVL, RVL and RVM in going up and down stair (r = 0.495, 0.548, and 0.554, respectively, P < 0.01).

## Discussion

This study reveals that during stair ascending, people with KOA display a notable change in quadriceps activation patterns, marked by heightened RMS amplitude of the VM and diminished MDF of the VL. These findings corroborate the recognized concept that KOA patients utilize compensatory neuromuscular procedures, engaging supplementary motor units and maintaining muscle activity to counteract joint instability and weakness, thus boosting RMS. The concurrent decrease in VL MDF indicates a greater susceptibility to muscle fatigue, emphasizing a specific deficit in muscular endurance within the KOA group. Significantly, our findings demonstrate task-specific relationships between JPR absolute error angles—a sign of proprioceptive impairment—and sEMG parameters of the quadriceps during ascending stairs, associations that were not present in the healthy controls. At greater knee flexion degrees, a rise in JPR error exhibited a positive correlation with VM RMS and a negative correlation with VM MDF. This suggests that an increase in knee flexion angle may lead to reduced proprioceptive acuity, which could require heightened activation of the vastus medialis to stabilize the joint. This compensatory mechanism may inadvertently result in increased muscular fatigue, potentially establishing a detrimental cycle. Stair negotiation for KOA patients represents not only a mechanical challenge but also highlights a notable impairment in the integration of proprioceptive input and muscular output within the sensorimotor system. This study used both the time-domain index RMS and the frequency-domain index MDF for analysis. RMS is used to determine the quadriceps femoris activation level. Considering the fact that the task in this study did not last long enough to cause considerable muscle fatigue, MDF was chosen as an indication based on the following criteria: MDF is not only sensitive to fatigue, but it also reflects muscle fiber type and action potential conduction velocity. Evidence suggests that patients with knee osteoarthritis have atrophy of the quadriceps fast-twitch muscle fibers as well as alterations in neuromuscular control techniques, which may result in variances in MDF baseline values (Jieting et al., 2024). As a result, evaluating MDF can enhance the activation ‘quantity’ information given by RMS in terms of muscle function ‘quality’, revealing the aberrant functional condition of the quadriceps femoris in KOA patients.

A significant observation is the varying association of muscle activity patterns across activities among different groups. In the KOA group, substantial positive correlations were noted for both RMS and MDF of the VL and VM during level walking and stair ascent/descent. This indicates that KOA patients use a relatively inflexible, task-invariant neuromuscular control technique. Impaired proprioceptive feedback and decreased joint stability likely obstruct the capacity of central nervous system to precisely regulate muscle activation patterns according to the specific requirements of various tasks (Marasco and de Nooij, 2023; Pan et al., 2023). As a result, a generalized and perhaps less efficient muscle co-activation pattern may be employed to sustain fundamental function. The healthy controls demonstrated inter-task consistency predominantly in the MDF of the lower limb muscles, indicative of intrinsic muscle physiological characteristics, but not in their RMS, which signifies immediate mobilization levels. This distinction emphasizes the ability of a healthy neuromuscular system to adapt muscle recruitment in response to task requirements while preserving stable muscle physiology (Chen et al., 2021). The persistent correlation of both RMS and MDF across tasks in KOA patients highlights a reduction in motor control flexibility, providing direct evidence of impaired sensorimotor integration—the diminished capacity to convert precise joint position sense into optimal, task-specific muscle activation commands (Ucurum et al., 2024).

Our data collectively corroborate the hypothesis that KOA extends beyond localized articular cartilage degeneration, signifying a systemic pathophysiological process that encompasses proprioception, neuromuscular control, and muscle function. The established correlations between JPR errors and sEMG parameters, along with the atypical task-invariance of muscle activation patterns, offer a cohesive framework for understanding functional impairment in KOA (Chen et al., 2023; Si et al., 2024; Alshahrani and Reddy, 2023). Proprioceptive distortion, evidenced by heightened JPR error, compromises the basis of joint stability feedback, resulting in inaccurate motor orders from the central nervous system. The muscular system, especially the quadriceps, compensates by exhibiting heightened activation amplitude and decreased efficiency. This maladaptive neuromuscular technique, frequently utilized in diverse weight-bearing activities, directly hinders tasks such as stair ascent and may, over time, worsen joint degeneration by elevating aberrant joint stresses. Consequently, framing KOA as a “neuromuscular arthritis,” in which muscle, nerve, and joint interact within a pathogenic triad, may be more appropriate (Hansen et al., 2024; Knoop et al., 2021; Lu et al., 2018).

The current findings may have clinical consequences. The evaluation of KOA patients should encompass not only pain and structural assessments but also sEMG parameters during daily activities and JPR at designated angles, as these metrics can more effectively identify deficits in neuromuscular control. Secondly, rehabilitation methods must aim to interrupt the detrimental cycle between proprioceptive deficits and abnormal muscle activation (Dhumale and Shinde, 2025; Liu et al., 2025; John Prabhakar et al., 2020). Our findings suggest that targeted therapies, including proprioception-focused training alongside exercises aimed at enhancing quadriceps activation patterns and delaying fatigue, may be more efficacious than conventional strength training in enhancing functional performance and potentially decelerating disease progression. Future research should concentrate on multiple avenues: utilizing longitudinal designs to substantiate the viability of sEMG and JPR parameters as biomarkers for KOA progression and rehabilitation effectiveness; investigating advanced neuromuscular re-education techniques that combine proprioceptive training with task-oriented practice; and employing more intricate analyses, such as muscle co-contraction indices, to enhance understanding of changes in multi-muscle coordination strategies in KOA.

## Limitations

The primary constraints of this study are as follows: The gender ratio among the subjects is skewed, leading to inadequate generalization of the research findings. The disparity may stem from the KOA group exhibiting an imbalance in the gender ratio, with middle-aged and elderly women demonstrating greater concern for the health of their sports joints and a higher propensity to engage in research initiatives. Then, the squat movement was employed as the RVC test movement, which differs from the conventional MVC measuring movement and may cause variations in the degree of normalization of sEMG data. The study employed a cross-sectional methodology, which could only demonstrate correlation but could not establish causative relationships or temporal sequences between aberrant muscle activity and proprioceptive abnormalities.

## Conclusion

This study analyzed sEMG signals and JPR errors in patients with KOA compared to healthy controls during stair climbing and walking, highlighting distinct changes in neuromuscular control and proprioception in KOA patients. The results indicate that during stair climb, KOA patients display a unique pattern of elevated RMS of the VM and reduced MDF of the VL. Notable relationships were identified between the absolute error angles of JPR and sEMG parameters of VM. The pronounced positive correlations in muscle activation patterns between walking and stair upward and downward activities indicate a fixed, task-invariant neuromuscular approach in KOA patients, possibly resulting from compromised sensorimotor integration due to proprioceptive impairments. These findings offer new insights into the pathogenesis of KOA from a neuromuscular-sensory viewpoint, emphasizing the need for future rehabilitation therapies to concurrently target proprioceptive accuracy and the enhancement of muscle activation patterns.

## Data Availability

The datasets presented in this study can be found in online repositories. The names of the repository/repositories and accession number(s) can be found below: DRYAD, https://doi.org/10.5061/dryad.fn2z34v8s.
